# Advancing Global Neurotrauma Surveillance Through National Registries: A Response to Recent Commentaries

**DOI:** 10.34172/ijhpm.2023.8288

**Published:** 2023-10-23

**Authors:** Ernest J. Barthélemy, Jacob Lepard, Anna E. C. Hackenberg, Joanna Ashby, Rebecca B. Baron, Ella Cohen, Jacquelyn Corley, Kee B. Park

**Affiliations:** ^1^Global Neurosurgery Laboratory, Division of Neurosurgery, SUNY Downstate Health Sciences University, Brooklyn, NY, USA.; ^2^Emory Children’s Center, Children’s Healthcare of Atlanta, Atlanta, GA, USA.; ^3^Department of Neurosurgery, Emory University School of Medicine, Atlanta, GA, USA.; ^4^Technical University of Munich, Munich, Germany.; ^5^Department of Anaesthesiology, LMU University Hospital, Ludwig-MaximilianUniversity of Munich, Munich, Germany.; ^6^School of Medicine, University of Glasgow, Glasgow, UK.; ^7^Department of Neurosurgery, Icahn School of Medicine at Mount Sinai, New York City, NY, USA.; ^8^Department of Internal Medicine, Icahn School of Medicine at Mount Sinai, New York City, NY, USA.; ^9^Samaritan Medical Group Brain & Spine Center, Corvallis, OR USA.; ^10^Program in Global Surgery and Social Change, Department of Global Health and Social Medicine, Harvard Medical School, Boston, MA, USA.

 We were delighted to read the six commentaries addressing our scoping review^1^ and comparative analysis of neurotrauma data dictionaries from the national registries of low- and middle-income countries (LMICs). The correspondents have expanded our consideration of the challenges and opportunities inherent to the use of national trauma registries as a vital tool in advancing neurotrauma surveillance. Herein, we endeavor to respond to the queries raised by the commentators, and highlight common themes raised by their thoughtful correspondences.

## Neurotrauma Surveillance in Latin America

 Rubiano and Clavijo highlight the importance of neurotrauma registry development for advancing organized neurotrauma care worldwide.^2^ We thank the authors for sharing their experience with the LATINO-TBI registry, a project that aspires to fill the regional gap in neurotrauma data from the LMICs of Latin America and the Caribbean. The barriers encountered in the implementation of the LATINO-TBI registry, ie, lack of administrative support, incomplete data on prehospital care and outcomes, and inadequate healthcare investment prioritization by governments and institutions committed to the sustainable development of these regions, are commonly encountered in other LMICs. A survey of prehospital and emergency leaders from 13 LMICs in Africa, Asia, and Latin America revealed significant heterogeneity in prehospital care services, general lack of prehospital care service development, and barriers that include inadequate funding, lack of leadership and absence of legislation setting standards.^3^ These concerns are echoed in a review by Bommakanti et al, citing lack of resources, insufficient prehospital care and challenges with administrative duties and hospital organization as the most significant barriers to successful trauma registry implementation in LMICs.^4^ Collectively, these challenges constitute key advocacy targets for trauma care clinicians and researchers.

## National vs. Institutional Trauma Registries

 Boeck et al offer constructive criticism of our work that highlights challenges to a presumed gold standard of global adaptation of national neurotrauma registries, while promoting the value of institutional registries.^5^ We are aligned with the pursuit of, “progress over perfection”; our work was specifically designed to inquire into the current state of national neurotrauma registry development in LMICs, while offering a perspective that neurosurgeons are uniquely positioned to advance in the global health advocacy space.^6,7^ We support the development and continued utilization of institutional trauma registries in LMICs, and the inclusion of neurotrauma data elements as possible in these registries to facilitate institutional trauma care quality improvement. Given our explicit interest in neurotrauma care from national healthcare policy standpoint, the use of institutional registries that are not aligned with a national standard was, however, outside of our scope of inquiry.

 We acknowledge the authors’ concern that advocacy for national neurotrauma registries, “…reinforces an obsession with an ideal that prevents instead of augments progress.”^5^ Indeed, the challenge of strengthening trauma care systems at the national health policy level is complicated by the heterogeneity of fragmented healthcare systems in many LMICs.^8^ Nonetheless, we assert that centering national neurotrauma registry development in LMICs invites their public health leaders to consider how trauma surveillance systems might be (re-)designed and implemented to advance progress in neurotrauma surveillance. The World Health Organization’s (WHO’s) Minimum Data set for Injury is a consensus-based set of core data elements recommended following extensive consultation with members of the international community as a standardized starting point from which individual institutions can add site specific variables at will, while still allowing for participation in a national registry.^9^ We therefore highlight this dataset as an inclusive, and adaptable data dictionary designed to support national trauma care objectives of healthcare governments in LMICs.

## Approaches to Trauma Registry Development

 Asfaw’s commentary recalls that the successes of global health policy advocacy in infectious diseases provide instructive templates for global surgery, trauma and emergency care.^10^ The author cites a Nigerian example of registry development for HIV-associated cancer utilizing the Research Electronic Data Capture system, which offers a ubiquitously available global resource that can readily be adapted by LMICs for trauma registry development.^10^ Several commentaries cited here note that the need for trained data management personnel remains a key hurdle to address, even when cost barriers are mitigated by free and open access technologies.^2,10-12^ The opportunities highlighted by successful registry implementation in LMICs, such as the examples in Uganda and Nigeria cited here, can strengthen the advocacy platform for increased government funding to establish these lacking, yet indispensable human resources for trauma registry development and implementation.^10,11^

 The commentary by Lecky reflects on the role of grassroots-level public health leadership and social entrepreneurialism.^13^ The author asserts that successful examples of national trauma registries are more commonly enterprises that were inspired by the need for data. Moreover, their implementation on a national scale does rely upon the establishment of multiple feedback loops between clinical traumatologists, the (paid) trauma or neurotrauma lead for the registry, hospital trauma audit meetings, and health care governance officers.^13^ We appreciate the author’s centering the third key question prioritized by the Global Emergency Care Research Network, an international collaboration composed primarily of representatives from LMICs: *“What are the obstacles to implementing emergency care/trauma registry-based systems in LMICs?”* We appreciate the author’s perspective that despite the limitations of our methods, the rigor and resulting findings of our approach emphasize the need for funding agencies to prioritize this concern.

## National Neurotrauma Surveillance Beyond Registries

 Schenck and Mangat offer recommendations regarding the broader aspects of national neurotrauma surveillance that must complement institutional-level data from hospital-based neurotrauma registries.^7^ They highlight many additional prospective sources of neurotrauma surveillance data, such as death records, police reports and various other community records already collected by stakeholders who might synergistically collaborate with their nations’ public health officers. We agree with the authors’ assertion that health care governments are accountable to protecting their citizens from all health threats, including road traffic accidents, violence, and other potential etiologies of neurotrauma. The bottom-up advocacy efforts recommended by the authors build upon our recommendations in a holistic manner that we trust will be useful to health ministries, and stakeholders in community healthcare and global neurotrauma alike.

 Thango et al highlight specific barriers to neurotrauma research capacity in LMICs, including an enormous burden of disease, lack of human and material resources, and contextual factors such as linguistic differences, social disparities, and barriers to data dissemination.^11^ However, the authors present an example from Uganda illustrating the feasibility of negotiating these barriers to implement an electronic record-based trauma registry.^11^ Recognizing the rapidly approaching 2030 deadline of the Sustainable Development Goals, we thank the authors and join them in renewing the call for clinician-led, data-driven policy advocacy that raises awareness of neurotrauma as a neglected source of national and global morbi-mortality.

## Ethical issues

 Not applicable.

## Competing interests

 Authors declare that they have no competing interests.
